# Genomic Investigation Reveals a Community Typhoid Outbreak Caused by Contaminated Drinking Water in China, 2016

**DOI:** 10.3389/fmed.2022.753085

**Published:** 2022-03-01

**Authors:** Bin Hu, Peibin Hou, Lin Teng, Song Miao, Lijiang Zhao, Shengxiang Ji, Tao Li, Corinna Kehrenberg, Dianmin Kang, Min Yue

**Affiliations:** ^1^Shandong Provincial Center for Disease Control and Prevention, Jinan, China; ^2^Department of Veterinary Medicine, College of Veterinary Medicine, Zhejiang University, Hangzhou, China; ^3^Shandong Medical College, Jinan, China; ^4^Linyi Center for Disease Control and Prevention, Linyi, China; ^5^Institute for Veterinary Food Science, Justus-Liebig-University Giessen, Giessen, Germany; ^6^State Key Laboratory for Diagnosis and Treatment of Infectious Diseases, National Clinical Research Center for Infectious Diseases, National Medical Center for Infectious Diseases, The First Affiliated Hospital, College of Medicine, Zhejiang University, Hangzhou, China; ^7^The Hainan Institute of Zhejiang University, Sanya, China

**Keywords:** *Salmonella* Typhi, outbreak, typhoid fever, drinking water, genomics

## Abstract

Typhoid fever is a life-threatening disease caused by *Salmonella enterica* serovar Typhi (*S*. Typhi) and remains a significant public health burden in developing countries. In China, typhoid fever is endemic with a limited number of reported outbreaks. Recently, Chinese local Center for Disease Prevention and Control is starting to apply whole genome sequencing for tracking the source of outbreak isolates. In this study, we conducted a retrospective investigation into a community outbreak of typhoid fever in Lanling, China, in 2016. A total of 26 *S*. Typhi isolates were recovered from the drinking water (*n* = 1) and patients' blood (*n* = 24) and stool (*n* = 1). Phylogenetic analysis indicated the persistence of the outbreak isolates in drinking water for more than 3 months. The genomic comparison demonstrated a high similarity between the isolate from water and isolates from patients in their genomic content, virulence gene profiles, and antimicrobial resistance gene profile, indicating the *S*. Typhi isolate from drinking water was responsible for the examined outbreak. The result of pulsed-field gel electrophoresis (PFGE) revealed these isolates had identical PFGE pattern, indicating they are clonal variants. Additionally, phylogeographical analysis of global S. Typhi isolates suggested the outbreak isolates are evolutionarily linked to the isolates from the United Kingdom and Vietnam. Taken together, this study highlights the drinking water and international travel as critical control points of mitigating the outbreak, emphasizing the necessity of regular monitoring of this pathogen in China.

## Introduction

*Salmonella enterica* contains >2,600 identified serovars and is commonly related to gastroenteritis in humans, threatening the public health system over the world (1). Human typhoid fever is a life-threatening illness caused by consuming food or water contaminated by *Salmonella enterica* serovar Typhi (*S*. Typhi) (2, 3). Though typhoid fever is rarely reported in developed countries, it has been recognized as a significant disease burden in the developing world (4). The estimated numbers of annual global incidence and death caused by typhoid fever were 11.8 million and 128 thousand, respectively (5, 6).

Typhoid fever is endemic and poses an increasing burden in China (7, 8). According to the Chinese national surveillance system, the total number of typhoid fever cases was 8,850 (0.65 per 100,000 people) in 2015 (8). The majority of them are sporadic cases, while the outbreak of typhoid fever is rarely reported (9, 10). In these sporadic cases, Pulse Field Gel Electrophoresis (PFGE) was widely applied to generate bacterial DNA fingerprints to trace the sources of *S*. Typhi causing typhoid fever, but the PFGE may cause misleading results because of its low resolution in distinguishing strains (11, 12). Compared with PFGE, whole-genome sequencing (WGS) data generates a higher resolution by using the complete DNA sequence of a bacterial genome, increasing the accuracy of source tracing (11). With a decreasing price for WGS, it has been applied as routine work in the public health agencies of numerous developed countries, e.g., Food and Drug Administration and Centers for Disease Control and Prevention in the United States and European Center for Disease Prevention and Control (13). However, only one study used WGS to track the source of typhoid fever outbreaks in China (9).

Identifying the transmission routes and geographical origins of clinical *S*. Typhi is critical for preventing and mitigating the outbreak and sporadic typhoid fever cases. *S*. Typhi can also transmit among countries through international travelers, and WGS is a powerful tool to identify their international transmission and investigate the geographical origin of these clinical isolates (9, 14). In China, typhoid fever incidence mainly occurred in Southern China provinces adjacent to South and Southeast Asia countries, suggesting bacterial transmission between China and South and Southeast Asia countries (8). However, very few Chinese *S*. Typhi isolates have been sequenced and archived in public databases, resulting in a poor understanding of their epidemiological nature and geographical origin. The newly generated genomic data would add value for further understanding of global transmission dynamics in *S*. Typhi.

The incidence rate of typhoid fever in North China is lower than that in South China (8), but a community-wide typhoid fever outbreak was reported in Lanling county, Shandong (North China) in 2016. In this study, we conducted WGS for outbreak investigation and aimed to identify the source and the geographical origin of the outbreak isolates by epidemiological investigation, comparative genomics, and phylogenetic analysis.

## Materials and Methods

### Ethical Statement

Blood and fecal samples from patients (*n* = 31) were obtained under a local surveillance program in accordance with Shandong Disease Control and Prevention (CDC) and local hospitals, following the recommendations of the Chinese Center for Disease Control and Prevention. Informed written consent for the use of surveillance samples was obtained from the patient.

### Epidemiological Investigation

Between January and May 2016, a suspected community outbreak of typhoid fever was reported in Lanling county, Shandong. At the end of May 2016, an epidemiological investigation was conducted by Shandong CDC to identify the source of the outbreak and the number of affected patients. The suspected cases were reported to Shandong CDC through an online disease reporting system. A case was defined as suspect typhoid fever when a patient had a sustained fever (≥38°C, for more than 3 days) accompanied with headache and fatigue but without other diagnosed cause of fever. A case of clinical typhoid fever was confirmed when *S*. Typhi was identified from the blood or stool of a person with suspect typhoid fever. To investigate the potential source of the outbreak, the patients who had suspected typhoid fever from January to May 2016 were visited for face-to-face interviews using a questionnaire by the epidemiological experts from Shandong CDC. The detailed patient information and exposure history focus on the age, gender, residential location, food and water consumption, and travel history.

### Sample Collection and Bacterial Identification

Before the investigation initiated by the Shandong CDC, the blood and fecal samples of the patients were obtained by the local hospitals from January to May 2016. The earlier typhoid cases were also reported by this sentinel hospital that collected samples from patients with various infectious diseases, however, the possible source samples, including food, were not available at this time. A total of 31 blood samples and 31 stool samples from patients (*n* = 31) with suspect typhoid fever were collected to investigate the presence of *Salmonella* using selective media. In addition, workers from the local hospital and CDC collected fecal samples (*n* = 31) from 31 patients in the hospital, water samples (*n* = 50) from pipes in patients' homes and the community, and food samples (*n* = 5) in patients' homes to identify the potential causative agent (i.e., *Salmonella, Shigella* spp., *Campylobacter jejuni., Vibrio parahaemolyticus, Vibrio cholerae*) of the outbreak based on protocols of Chinese standard diagnostic criteria for infectious diarrhea (i.e., WS271-2007, WS287-2008, and WS289-2008) as previously described (15–18). The five food samples and fifty water samples were collected by CDC after May 26, 2016, the date when the investigation was started. Twenty-five grams of food sample was added to 225 ml of Buffered Peptone Water (BPW) for homogenization. The water sample (50 ml) was centrifuged at 12,000 × g for 30 min and removed the supernatant. To identify *Salmonella* from samples (e.g., feces, blood, water, and food), 1 g of fecal sample and 5 ml of blood sample (or 5 ml of water sample, or 5 ml of homogenized food samples) were inoculated into 10 and 50 ml of Tetrathionate Broth (TTB), respectively, with incubation at 37°C for 8 h, followed by streaking 100 μl of bacterial culture on Bismuth Sulfite (BS) agar with incubation at 37°C for 18 h to select candidates of *Salmonella* colonies with black or green color. The suspected *Salmonella* isolates were picked to confirm bacterial species using matrix assisted laser desorption ionization-time of flight mass spectrometry (MALDI-TOF MS). All the confirmed *Salmonella* isolates were stocked in 25% glycerol in a deep freezer at −80°C. When the CDC started the investigation, *invA* gene PCR (forward primer: 5′-GTGAAATTATCGCCACGTTCGGGCAA-3′; reverse primer: 5′-TCATCGCACCGTCAAAGGAACC-3′) was conducted for these isolates to confirm they are *Salmonella* using the following conditions: 95°C for 5 min; 95°C for 30 s, 64°C for 30 s, and 72°C for 30 s for 30 cycles; 72°C for 10 min (19) Twenty-five confirmed isolates from 25 patients were selected for whole genome sequencing.

### Pulsed-Field Gel Electrophoresis Analysis

Pulsed-field gel electrophoresis (PFGE) analysis is our routine work for investigating the genetic relatedness of outbreak isolates, therefore we conducted PFGE for *S*. Typhi isolates as previously described (20). Briefly, bacterial genomic DNA of 26 *S*. Typhi isolates from this outbreak were extracted using Wizard Genomic DNA Purification Kit based on manufacturer's protocol and digested by *Xba*I, followed by resolving fragment DNA using a CHEF Pulse-Field Electrophoresis System. A *S*. Typhi isolate 98,053 that was unrelated to this outbreak was used as the quality control for PFGE.

### Antimicrobial Susceptibility Testing

To test the antimicrobial susceptibility of the isolates, their minimal inhibitory concentrations (MICs) of 20 antibiotics were tested by the broth microdilution method using Mueller-Hinton broth. The MIC assay was conducted according to the guideline of the Clinical Laboratory Standards Institute (CLSI 2019) (21). The antibiotics used in the MIC assay contain Ampicillin (Amp), Cefotaxime (Ctx), Ceftazidime (Ctz), Cefepime (Cfp), Cefoxitin (Cxt), Imipenem (Imp), Aztreonam (Azt), Meropenem (Mer), Amoxicillin/Clavulanic acid (Amo/Cla), Chloramphenicol (Chl), Sulfamethoxazole/Trimethoprim (Sul/Tri), Nalidixic acid (Nal), Ciprofloxacin (Cip), Levofloxacin (Lev), Amikacin (Ami), Gentamicin (Gen), Kanamycin (Kan), Azithromycin (Azi), Doxycycline (Dox), and Tetracycline (Tet). The standard strain *E. coli* ATCC 25922 was used for quality control in the MIC assay.

### DNA Extraction and Whole-Genome Sequencing Analysis

The DNA of *S*. Typhi isolates (*n* = 26) were extracted from their overnight cultures using Wizard Genomic DNA Purification Kit according to the manufacturer's protocol, followed by constructing their DNA libraries. The DNA libraries were sequenced using Illumina HiSeq X Ten to acquire pair-end reads of 150 bp. These raw reads were trimmed by Trimmomatic (Version 0.38) (22) and assembled using SPAdes (Version 3.0) (23). The assembled genomes were annotated using NCBI Prokaryotic Genome Annotation Pipeline (24).

### Whole-Genome Comparison of *S*. Typhi Isolates During the Outbreak

The sequence types of *S*. Typhi isolates were identified by uploading their genomic sequences to MultiLocus Sequence Typing (MLST) Center in Genomic Epidemiology (https://cge.cbs.dtu.dk/services/). The subclade of these isolates was genotyped based on 68 single nucleotide polymorphisms (SNPs) by mapping their sequence to the reference strain CT18 (accession number AL513382) using Parsnp (HarvestTools version 1.2) (14, 25). The vcf file generated by Parsnp was used to identify the SNP loci in each isolate and the subclade of each *S*. Typhi isolate was determined by its SNP pattern (14). Virulence factor genes were identified by aligning bacterial genomic sequences against the reference sequences in Virulence Factors Database (VFDB) using BLASTn (26). The query sequences with ≥80% identity and ≥80% coverage were identified as virulence factor genes. The antimicrobial resistance genes in these isolates were identified by ResFinder 4.0 (27). The presence of plasmid was identified using plasmidFinder 2.1 (28). As previously described, the genomic DNA sequences of 26 isolates were concatenated and compared using BLAST Ring Image Generator (BRIG), with isolate 1114 as a reference (29, 30).

### Phylogenetic Analysis

Phylogenetic analyses were conducted using different methods as previously described (20, 31). To understand the relatedness of 26 isolates from this outbreak, we first generated a core genome-based phylogenetic tree containing these isolates using Parsnp (HarvestTools version 1.2) (25). The CT18 (accession number AL513382) was used as a reference strain. Parsnp detected and removed the recombination regions by PhiPack and constructed a maximum-likelihood phylogenetic tree using FastTree2 (32, 33). Based on the result of the phylogenetic analysis, the isolate 681 was the closest to the outgroup strain CT18 in the phylogenetic tree. Therefore, we re-generated a core genome-based phylogenetic tree containing only 26 outbreak strains from this study using the same method and rooted the tree to the isolate 681 (reference and outgroup isolate). To investigate the geographical origin of the outbreak isolates, genomic sequences of the isolates from this study (*n* = 26) and ST2 *S*. Typhi isolates from Enterobase (*n* = 2,437, http://enterobase.warwick.ac.uk/, accessed 3 February 2021) were downloaded to construct a core genome-SNP based phylogenetic tree. The sequences of these isolates were mapped to the complete genomic sequences of a reference strain Ty2 (accession number AE014613) using Snippy (34). The core genome-SNPs were extracted using Snippy to generate a maximum-likelihood phylogenetic tree using IQtree with 1,000 bootstrap replicates (34, 35). The tree was rooted to Ty2 that belonged to ST1 and was visualized using iTOL (36). The vcf files generated by Parsnp and Snippy were used to calculate the number of pairwise SNPs using an in-house script.

### Accession Number(s)

All the sequencing data of the *S*. Typhi isolates from this study have been deposited in GenBank under BioProject no. PRJNA686895 (https://www.ncbi.nlm.nih.gov/bioproject/PRJNA686895). The accession number of bacterial sequences were listed in Supplementary Table S1. Publicly available software were used for all bioinformatics analysis conducted in this study.

## Results

### Typhoid Fever Epidemic in a Community in Lanling

**Figure 1:**
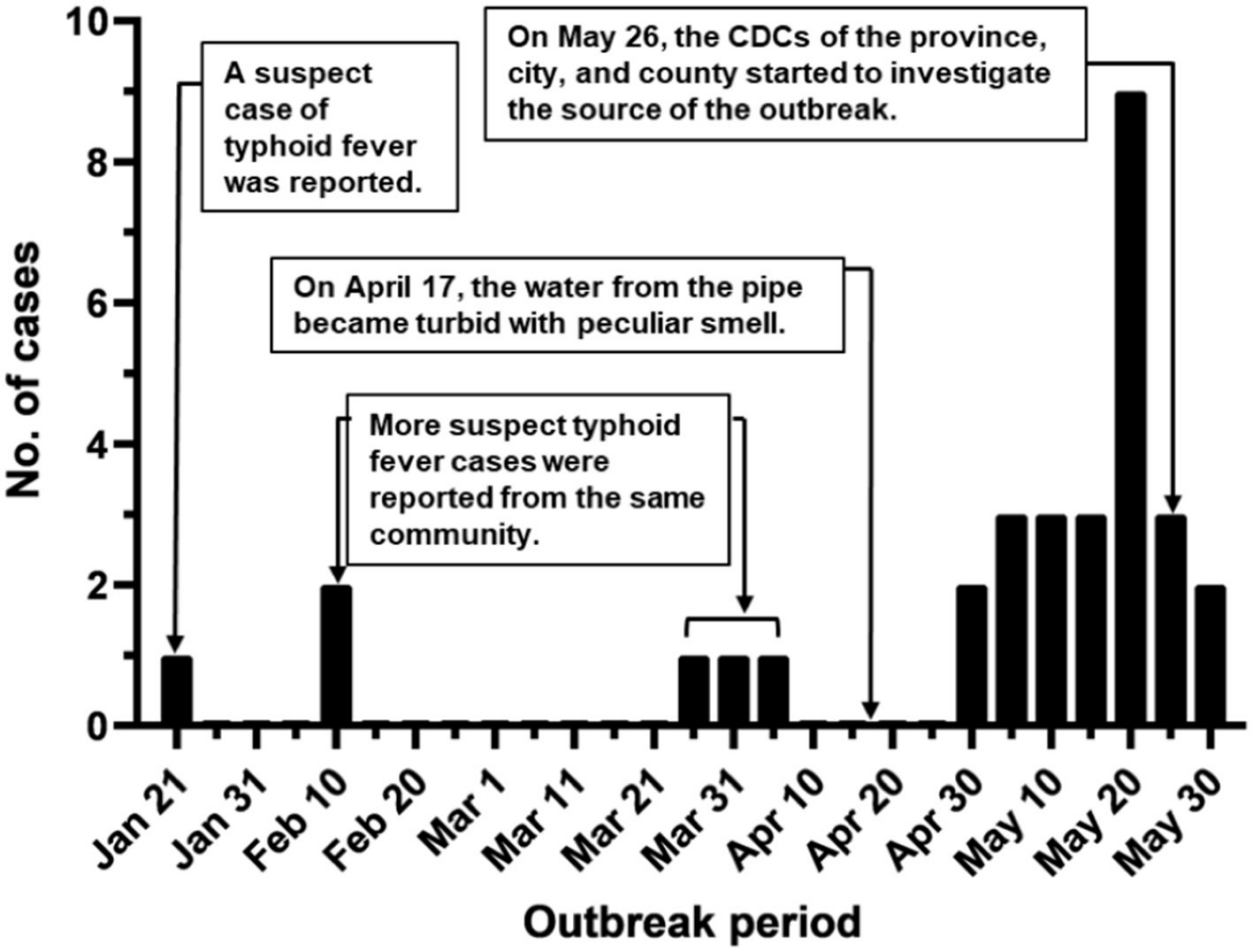
Outbreak period of typhoid fever. Typhoid cases were documented during the outbreak in Lanling, China, in 2016.

During January-May 2016, a suspected community-wide *S*. Typhi outbreak was observed in Lanling county of Shandong, China. The first documented case was a 42-year-old male with fever (≥40°C) for 10 days starting from Jan-21 (Figure 1). Hereafter, sporadic cases from the same community were reported between February and March (*n* = 5). An increasing number of patients was detected in late April and May (*n* = 25), with a peak of nine cases per day. By the end of May, 31 suspect cases of typhoid fever were reported from the same community. Among these cases, 14 patients (45.2%) were male, and 17 of them were female (54.8%). The ages of the patients ranged from 10 months to 55 years, with a median age of 22 years. These patients included 13 children under 10 years, 4 adolescents between 11 and 20 years, 4 youth at the age 21-30, 5 people between 41 and 40 years, and 3 patients elder than 51. Screening for potential causative agents by PCR revealed that only *Salmonella* was present in 25 out of 31 patients (24 blood samples and 1 fecal sample). The result of pathogen detection and the patients' syndromes indicated *S*. Typhi was the causative agent.

### Epidemiological Investigation of the Potential Source of *S*. Typhi

On May-26, 2016, CDC started a survey, including questionnaires for patients, to identify the source of the *S*. Typhi. As this outbreak only occurred within the same community, food and water were likely the sources of *S*. Typhi, which caused the outbreak. Though foods were reported as sources of *S*. Typhi (9), no evidence from the questionnaires suggested the patients consumed the same foods, indicating *S*. Typhi causing the outbreak may not be associated with the foods. However, the drinking water was reported as low-level turbidity during the outbreak. Therefore, we investigated whether the drinking water in this community plays a role in the examined outbreak. According to our survey, the drinking water supplied to this community was from a deep well, pumped to a water tower for storage, and delivered to each apartment without any sterile process. The water pipes in this community were used for more than 10 years and sporadically corroded, resulting in the leakage of water from pipes. Additionally, some water pipes were closed to septic tanks or merged by wastewater, suggesting the drinking water in pipes might be plausibly contaminated.

### Laboratory Investigation

**Figure 2:**
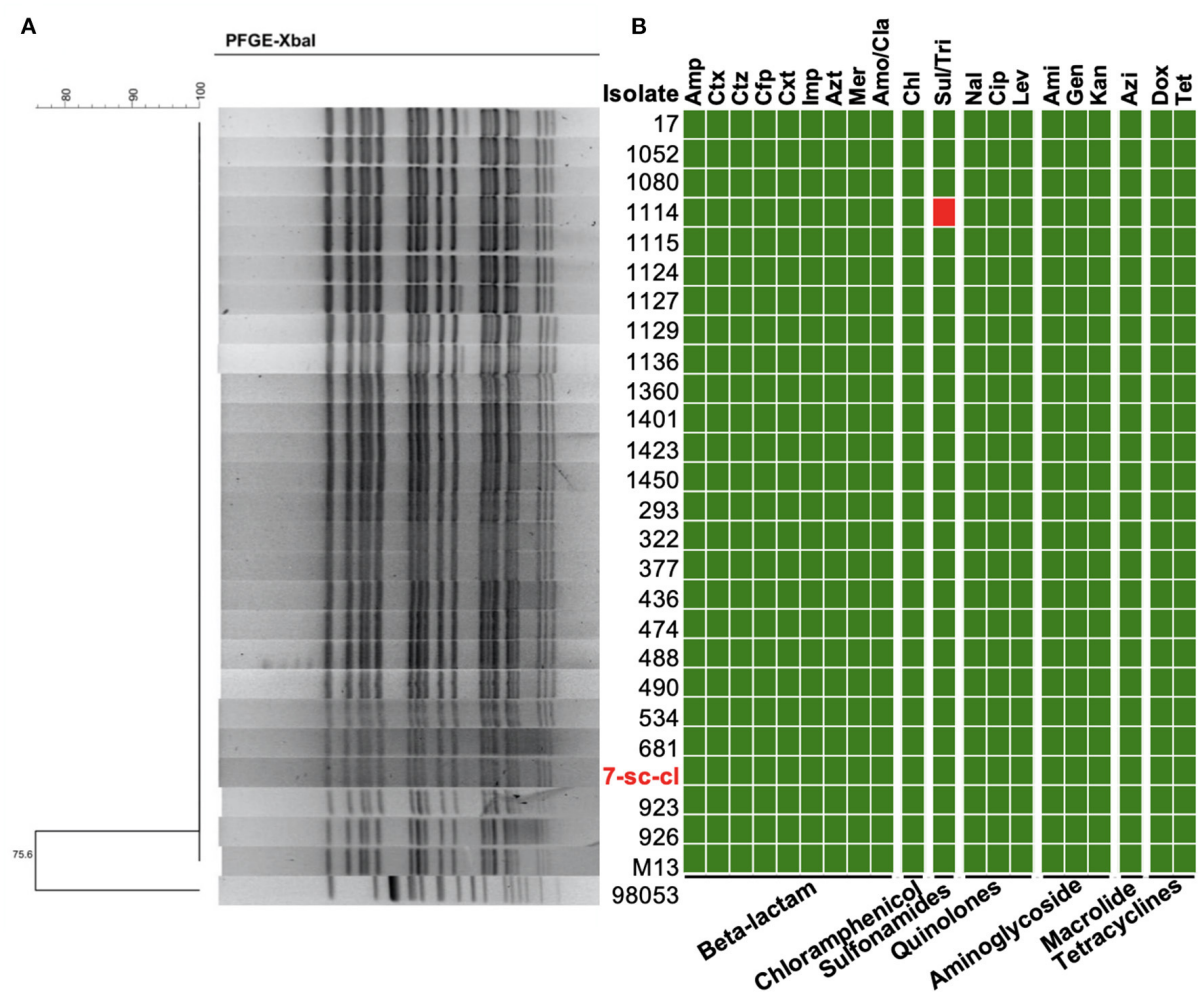
Molecular characterization of *S*. Typhi outbreak isolates. **(A)** PFGE patterns of the *S*. Typhi isolates digested with *Xbal*I. PFGE was conducted using 26 isolates in this study and a *S*. Typhi strain not related to this outbreak (98,053) was used as the control. **(B)** Minimum inhibitory concentration of the outbreak isolates. MICs of 20 antimicrobials belonging to seven antimicrobial classes were tested. The resistance (red block) and susceptibility (green block) of isolates to the antibiotics were indicated using different colors.

To track the source of *S*. Typhi in this outbreak, water from the water tower and pipes (*n* = 50), as well as food samples (*n* = 5) in patient's homes, were collected to detect the presence of this bacterium. The prevalence of *S*. Typhi was 2% (1/50) in the water sample and 0% (0/5) in the food sample. A *S*. Typhi isolate (7-sc-cl) was recovered from drinking water in a pipe, suggesting a potential role of waterborne transmission for this *S*. Typhi outbreak. Pulsed-field gel electrophoresis (PFGE) generates fingerprints for bacteria and is routinely applied to trace the source of the outbreak isolates (12, 37). The result showed that all 26 outbreak isolates (i.e., 25 patient isolates and 1 water isolate) have an identical PFGE pattern (Figure 2A), suggesting these isolates may be clonal variants, and *S*. Typhi from water is responsible for this outbreak.

Multidrug-resistant bacterial pathogen causes antimicrobial treatment failure, threatening public health. To understand whether these isolates are multidrug-resistant, we investigated their antimicrobial susceptibility profiles by determining the minimal inhibitory concentration (MIC) of 20 antibiotics belonging to seven antibiotic classes. Except for the isolate 1114, the other 25 isolates were susceptible to all tested antibiotics (Figure 2B and Supplementary Table S2). The isolate 1114 is resistant to sulfamethoxazole/trimethoprim.

### Phylogenetic Relatedness of *S*. Typhi Isolates Recovered From This Outbreak

**Figure 3:**
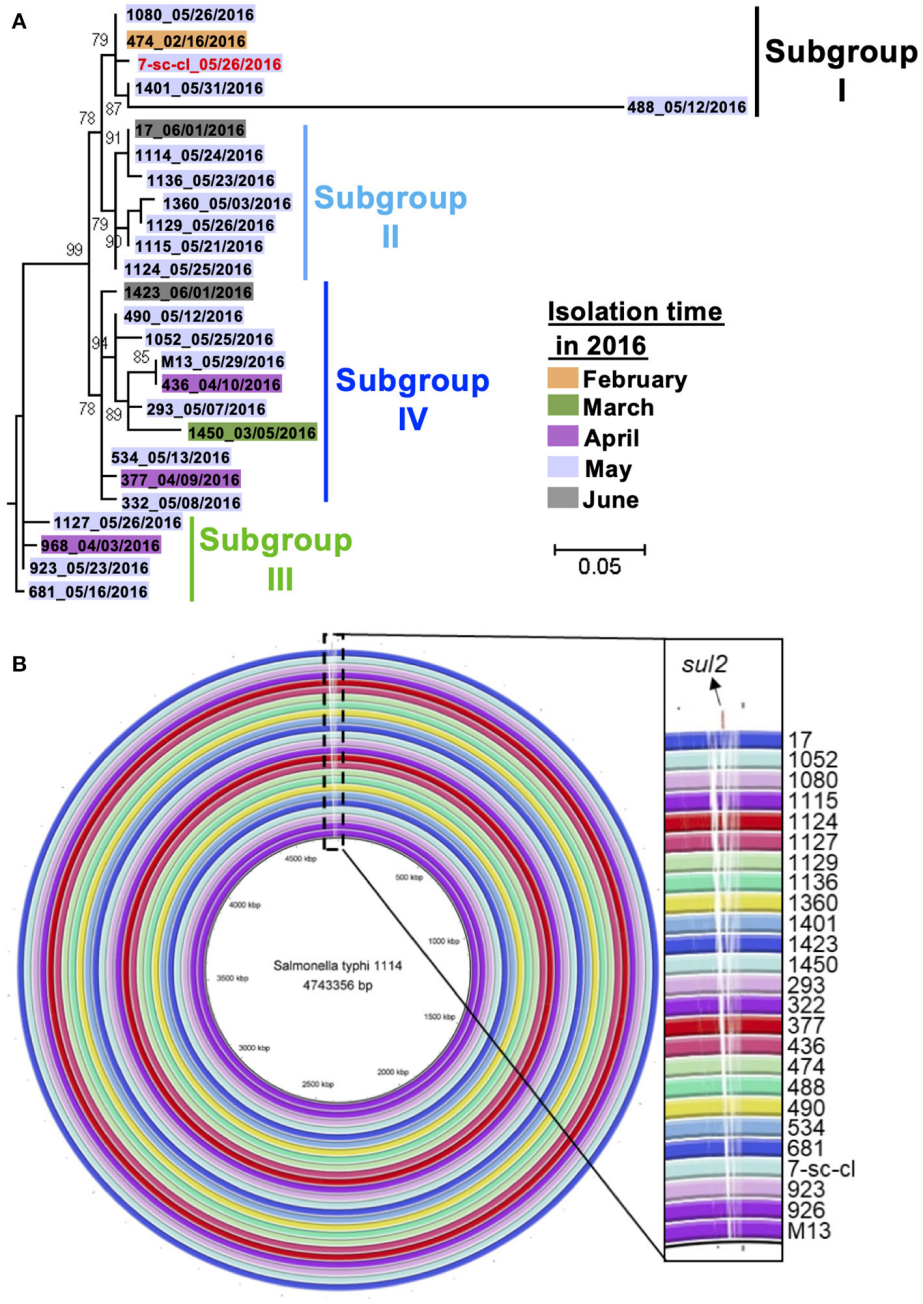
Relatedness of *S*. Typhi outbreak isolates. **(A)** Phylogenetic relatedness of *S*. Typhi outbreak isolates in this study. A maximum-likelihood phylogenetic tree of 26 *S*. Typhi isolates was generated using Parsnp based on core-genome SNPs. These isolates are from patients' stools (*n* = 25, black color) and a water sample (*n* = 1, red color). **(B)** Whole-genome alignment of 26 *S*. Typhi outbreak isolates. The genomic sequences of 25 isolates were aligned to that of the reference strain, i.e., 1114, by BRIG. The white blocks in each ring indicate the regions are absent in query isolates but present in the reference strain.

Whole-genome sequencing (WGS), with a higher resolution than PFGE, was applied to confirm the phylogenetic relationship of these isolates, as PFGE has certain limitation in distinguishing genetic differences between phylogenetically closed isolates (38). Therefore, we sequenced the whole genome of 26 isolates using Illumina HiSeq for phylogenetic analysis. Their genomic sequences range from 4,724,342 to 4,793,859 bp (Supplementary Table S1). A maximum-likelihood phylogenetic tree was constructed based on extracted SNPs in the core genome of 26 isolates using Parsnp (Supplementary Figure S1). The core genome of these isolates consisted of 98% of their whole genome, with 54 total SNPs identified. All these isolates clustered into four suggested subgroups which contain small numbers of pairwise SNPs within each subgroup, i.e., ≤ 32 pairwise SNPs within subgroup I, ≤ 4 pairwise SNPs within subgroup II, ≤ 3 pairwise SNPs within subgroup III, and ≤ 7 pairwise SNPs in subgroup IV (Figure 3A and Supplementary Figure S1). Interestingly, except for the isolate 488, the other four isolates from subgroup I (i.e., a water strain and three patient isolates) have ≤ 2 pairwise SNPs, indicating these four isolates are clonal variants. Importantly, the water isolate (7-sc-cl) was obtained from May by a retrospective study, and all the isolates from the patient were recovered between February and May, suggesting the water isolate (7-sc-cl) could persist in water for more than 3 months and play a continuing role in infecting humans within the same community for over several months. The data further indicate that the water isolate may be one of the sources of this outbreak. If a typhoid outbreak was caused by a single source, the outbreak isolates would display a limited number of SNPs (e.g., <17 SNPs) and form a single branch (39). Considering the low mutation rate of *S*. Typhi (~0.63 SNP per genome per year) (40), the identification of a maximum of 42 pairwise SNPs and four phylogenetic subgroups between the isolates within only a few months indicates either there were multiple sources of the outbreak or a single source with genetically diverse co-existing variants (Supplementary Table S3). Together, these data demonstrated that the *S*. Typhi outbreak was originated from the contaminated drinking water in the pipes, as well as other potential sources, and water supply system within the community facilitated the bacterial dissemination, resulting in this typhoid outbreak.

### Genomic Characterization of the Outbreak *S*. Typhi Isolates

To understand the genetic makeup of these *S*. Typhi isolates, we investigated the genomic characteristics of these isolates by identifying their sequence type (ST), subclade, plasmid type, virulence genes, and antimicrobial resistance genes (ARGs). All these isolates were identified as ST2 by MultiLocus Sequence Typing (MLST). The subtype of *S*. Typhi was associated with geographical location and was used to predict their geographical source (14). By identifying the SNPs between the outbreak isolates and reference strain, CT18, we found that the outbreak isolates belong to subclade 3.2.1, which was detected in Thailand in the 1970s and 1990s (41). To understand the virulence of these isolates, we identified their virulence associated genes. All the isolates contain 104 VF genes, including *cdtB* (cytolethal distending toxin subunit B), and showed the identical virulence gene profile (Supplementary Figure S1). To understand whether these isolates contain ARGs, their genomic sequences were aligned with the reference sequences in Resfinder. Only the isolate 1114 contains a ARG, i.e., *sul2* gene which confers bacterial resistance to sulfonamides antibiotics, suggesting this isolate may acquire the *sul2* gene through horizontal gene transfer during the outbreak period. Interestingly, no plasmid was identified from any isolate by plasmidFinder, indicating the *sul2* gene in the isolate 1114 is not carried by the plasmid. Additionally, we compared the genomic architectures of these isolates by aligning their chromosomal DNA sequences using BRIG. All these isolates were highly similar in terms of their genomic sequences; however, the isolate 1114 showed unique regions which were absent in the remaining 25 isolates (Figure 3B). These unique regions contain *sul2* gene, transferase-encoding genes, and prophage-related genes, suggesting this isolate may acquire *sul2* gene through transposon-mediated horizontal gene transfer.

### Potential Geographical Origin of the Outbreak Isolates

**Figure 4:**
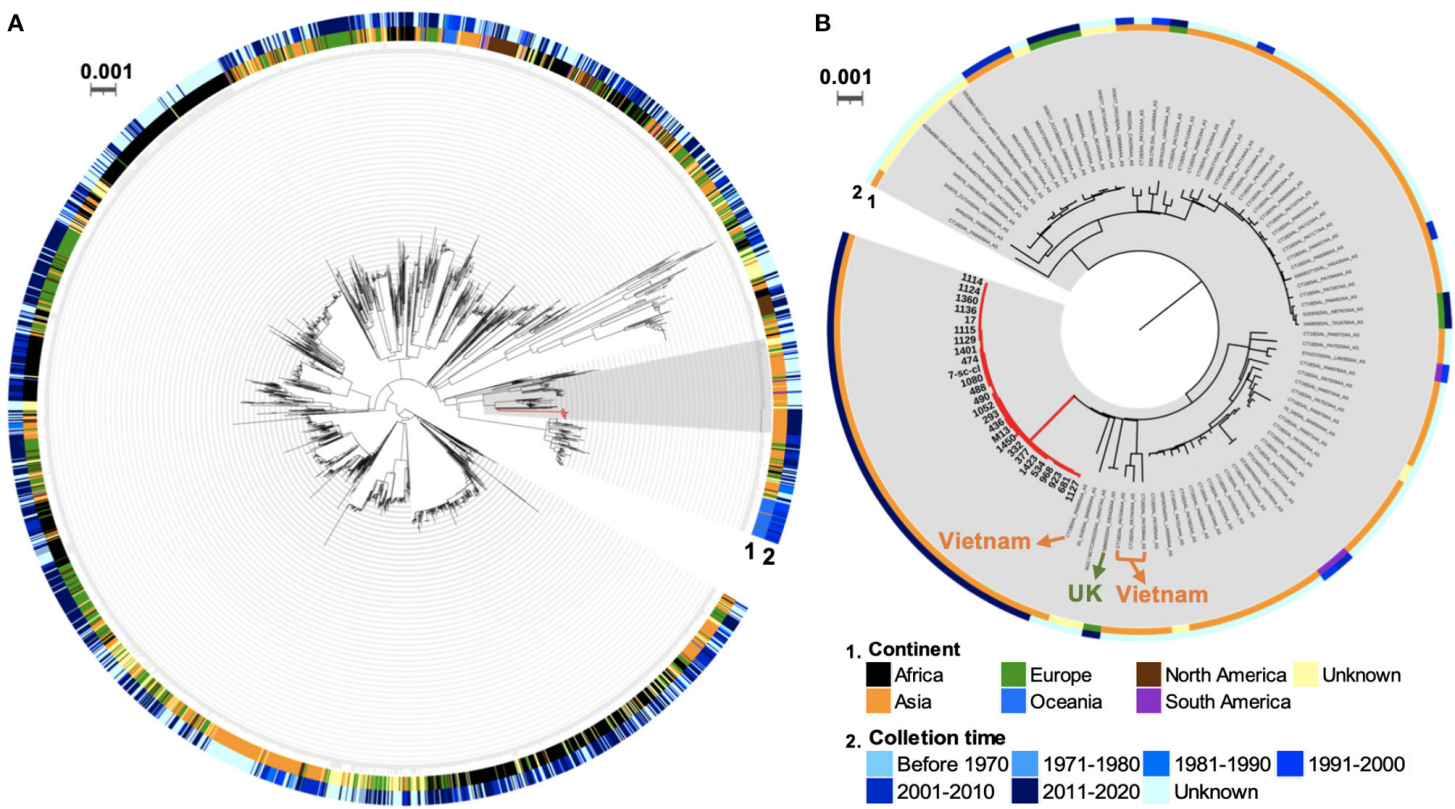
Global phylogenetic tree of *S*. Typhi ST2 isolates. **(A)** Phylogenetic relatedness of global *S*. Typhi ST2 isolates. **(B)** A subtree of global isolates with close relatedness to the outbreak isolates. The branches in red indicate the outbreak isolates in this study. Two rings represent the geographical origin (continent) and collection time of these isolates.

Transmission of *S*. Typhi between countries was observed because of global travelers who carried these isolates (14). To investigate the geographical origin of these *S*. Typhi ST2 outbreak isolates from this study, we generated a core genome SNP-based phylogenetic tree of 2,464 global *S*. Typhi isolates based on the diversity of 22,989 core-genome SNPs (Supplementary Table S4). This tree contains all 26 ST2 isolates from this study, 2,437 publicly available ST2 isolates from Enterobase, and a ST1 strain, Ty2, as an outgroup (Figure 4A). The outbreak isolates in the current study belong to a clade containing 104 isolates from Asia, Europe, South America, and unknown places. Among this clade, all 26 outbreak isolates formed a monophyletic cluster without any global strain (Figure 4B). Interestingly, these outbreak isolates clustered with a strain from the U.K., i.e., 588932|SAL_EB3426AA_AS (BioSample: SAMN10087161), and four isolates from Vietnam, i.e., CT18|SAL_PA6955AA_AS (SAMEA4425876), CT18|SAL_PA7045AA_AS (SAMEA4425974), CT18|SAL_PA7043AA_AS (SAMEA4425972), and CT18|SAL_PA7038AA_AS (SAMEA4425966). These isolates were split from the same node into distinct and long branches, suggesting the outbreak isolates in this study may share the same ancestor with the U.K. and Vietnam isolates and underwent local adaptation in China. As all patients in this study did not have a recent international travel history to the U.K. or Vietnam, the direct transmission from these two countries could be excluded. Taken together, these data indicate phylogenetic relatedness between the outbreak isolates and *S*. Typhi isolates from the U.K. and Vietnam.

### Intervention and Termination of the Typhoid Outbreak

According to the results of the epidemiological study, drinking water in the pipes was proposed as a source of *S*. Typhi that caused the outbreak. Hence, the water pipes were renewed, and the new water pipes were kept away from the septic tank or wastewater. To eliminate the risk caused by microbe from deep well water, taping water generated by a water plant was delivered to each apartment to replace the deep well water. All these actions ensured no more typhoid cases were reported after June 2016 and terminated this community-wide typhoid outbreak.

## Discussion

In the current study, we investigated a laboratory-confirmed, community-wide typhoid outbreak in Lanling county, China, between January to May 2016. Epidemiological investigation revealed a link between drinking water and the outbreak. The *S*. Typhi from drinking water and patients shows the same PFGE pattern, highly similar genomic contents, and close phylogenetic relatedness with small SNP numbers (0-42 SNPs). The genomic evidence, in combination with the epidemiological investigation, demonstrated that the *S*. Typhi from drinking water is the source of the outbreak. Phylogenetic analysis indicates the outbreak isolates are evolutionarily linked to the isolates from Vietnam and the U.K. The outbreak was immediately terminated after the replacement of water pipes and improving the hygienic environment of the community.

This community-wide typhoid outbreak is one of the biggest in Shandong province in recent years. The 4-month duration of this outbreak (Figure 1) indicates that the surveillance system was ineffective and the response was slow at the early stage. There might be a couple of reasons. Firstly, the community in this study is located in a rural area, where most of the patients preferred to visit private clinics rather than public hospitals. As the private clinics didn't report these cases, the outbreak was not monitored appropriately. Besides, Northern China generally showed a lower incidence rate of typhoid cases than Southern China (10). Due to the low incidence rate, the surveillance network for identifying typhoid cases in Northern China, e.g., Lanling county, was not well established at that time. Collectively, it is necessary to strengthen the surveillance system, especially in the rural areas and private clinics, to improve the public health reporting system in the whole China.

In this study, clonal variants of *S*. Typhi were identified from patients and drinking water in pipes, indicating the water is a source or vehicle for *S*. Typhi dissemination (Figure 3). Similarly, recent studies identified *S*. Typhi from water samples in many developing countries, e.g., Nepal and Indian, during typhoid outbreaks (42, 43). These water samples were either contaminated by feces or waste from the sewage drainage system (42, 43). This data indicated that drinking water is a critical control point to prevent typhoid outbreaks. Besides, although the water tower in the community was washed during the last 10 years, no disinfection measures were taken before this outbreak. The lack of disinfection and hygiene maintenance for the water tower may be another reason for this community-wide *S*. Typhi outbreak. Additionally, the water pipes in this study were broken and placed in close proximity to a septic tank, posing another risk factor for contamination. The total number of coliform bacteria and *E. coli* in some water samples from pipes exceeded the health standard of China (data not shown), supporting the contamination of water is the cause for this outbreak (44–46). Water pipe leak was a plausible reason to cause a recent typhoid outbreak in India (43), underlining the importance to ensure the entireness of pipes. Moreover, except for *S*. Typhi, other *Salmonella* serovars (i.e., Enteritidis and London) were detected in patients' stools (data not shown). Though these *Salmonella* Enteritidis and *Salmonella* London isolates were not directly identified in water samples in the current study, they may be also originated from the contaminated water. Therefore, disinfecting the water tower, maintaining the damaged pipes, and placing the water pipes in a sanitary location, e.g., places away from the septic tank and sewage drainage system, are essential control points to prevent the waterborne outbreak.

The WGS-based method provides a higher discriminatory power than the PFGE-based method in terms of bacterial genomic characterization and source tracing during a bacterial outbreak (11), even though all 26 outbreak isolates in this study cannot be distinguished by their PFGE pattern (Figure 2A). However, precise difference in their genomes can only be identified by using the WGS-based method (Figures 3A, B). The *sul*2 gene conferring bacterial resistance to sulfonamides antibiotics only presents in the chromosome of 1 isolate (i.e., the isolate 1114). Similarly, a recent study identified the *sul2* gene carried by transposons in the chromosome of *S*. Typhi isolates (40). As the microbiota of the human gut is a reservoir of ARGs, the mobile genetic elements in gut commensal bacteria show a potential to transfer ARGs, including *sul2* gene, to pathogens (47–49). Interestingly, the isolate 1114 in this study may acquire the *sul*2 gene from other antibiotic-resistant bacteria in the human microbiota through horizontal gene transfer. Nevertheless, we cannot rule out the possibility that the isolate 1114 acquired the *sul2* gene before it infected the patient. Most of the isolates recovered from this outbreak were susceptible, indicating a low level of antimicrobial resistant *S*. Typhi in general.

The global dissemination of *S*. Typhi, due to the carriage of this bacterium by international travelers, is an ongoing problem over the world (14). Similarly, a recent study proposed the association for the *S*. Typhi outbreak isolates between India and Xinjiang, China (9). All the outbreak isolates in this study belong to *S*. Typhi ST2 and subtype 3.2.1, which was previously reported in Thailand during 1970s and 1990s (41). According to the phylogenetic analysis, the outbreak isolates of this study and those isolates from the U.K. and Vietnam might have a common ancestor. Although there are a few subtype 3.2.1 isolates recovered from the U.K. and Vietnam, it is difficult to determine whether the patients had acquired these organisms during international travel. Due to very limited genomic data available and rarely available whole-genome sequencing investigations of Chinese *S*. Typhi isolates, key knowledge, regarding major subtypes or lineages, disease prevalence, and dynamic evolutionary history, remains unknown. Therefore, WGS routine surveillance of *S*. Typhi isolates, as well as genomic data from a historical collection, is the priority to address those questions, and possibly identify the burden of typhoid fever in China.

## Conclusion

In the current study, we identified drinking water as the source of the typhoid outbreak by WGS, emphasizing drinking water as a critical control point to prevent and mitigate the typhoid outbreak. Accordingly, it is necessary to ensure the entireness of water pipes for sanitary drinking water and conduct active surveillance of drinking water in communities with poor water supply systems.

## Data Availability Statement

All the sequencing data of the *S*. Typhi isolates from this study have been deposited in GenBank under BioProject no. PRJNA686895 (https://www.ncbi.nlm.nih.gov/bioproject/PRJNA686895).

## Ethics Statement

The studies involving human participants were reviewed and approved by the Chinese Center for Disease Control and Prevention. Written informed consent to participate in this study was provided by the patients/participants.

## Author Contributions

BH, PH, SM, LZ, SJ, TL, and DK collected the samples and conducted the survey. LT, BH, and PH conducted the experiments and analyses. LT wrote the draft of the manuscript. LT and MY finalized the manuscript. All authors contributed to the article and approved the submitted version.

## Funding

This material is based on work that is supported by the medical and health technology development program in Shandong (2017WS455), Zhifei disease prevention and control technology program of the Shandong Preventive Medicine Association (LYH2017-03), Chinese Important National Science and Technology Specific Projects (2018ZX10713001-002; 2018ZX10713003-002), National Program on Key Research Project of China (2019YFE0103900; 2017YFC1600103) as well as European Union's Horizon 2020 Research and Innovation Programme under Grant Agreement No. 861917– SAFFI, Zhejiang Provincial Natural Science Foundation of China (LR19C180001), and Opening Fund of Key Laboratory of Microorganism Technology and Bioinformatics Research of Zhejiang Province (2017E10010).

## Conflict of Interest

The authors declare that the research was conducted in the absence of any commercial or financial relationships that could be construed as a potential conflict of interest. The reviewer BZ declared a shared affiliation with several of the authors, MY and LT, to the handling editor at the time of review.

## Publisher's Note

All claims expressed in this article are solely those of the authors and do not necessarily represent those of their affiliated organizations, or those of the publisher, the editors and the reviewers. Any product that may be evaluated in this article, or claim that may be made by its manufacturer, is not guaranteed or endorsed by the publisher.

## Supplementary Material

Supplementary Figure S1 Unrooted phylogenetic tree of the examined isolates from this study. The maximum-likelihood phylogenetic tree was generated using Parsnp and visualized using iTOL.

Supplementary Figure S2 Virulence factor gene profiles of 26 *S*. Typhi isolates. The orange and red blocks indicate the isolates are from patients' stools and water samples, respectively. The blue blocks indicate the presence of the virulence genes.

Supplementary Table S1 The single nucleotide polymorphism matrix between Salmonella Typhi isolates.

Supplementary Table S2 The minimum inhibitory concentrations of 20 antimicrobials.

Supplementary Table S3 The SNP matrix of 26 outbreak-associated Salmonella Typhi isolates.

Supplementary Table S4 Meta information of 2,464 isolates used for global Salmonella Typhi ST2 phylogeny analysis.

Supplementary Table S5 Meta Information of 104 isolates belonging to the sub-tree of global Salmonella Typhi ST2 phylogenetic tree.
