# Corrigendum: Genomic Investigation Reveals a Community Typhoid Outbreak Caused by Contaminated Drinking Water in China, 2016

**DOI:** 10.3389/fmed.2022.918129

**Published:** 2022-05-09

**Authors:** Bin Hu, Peibin Hou, Lin Teng, Song Miao, Lijiang Zhao, Shengxiang Ji, Tao Li, Corinna Kehrenberg, Dianmin Kang, Min Yue

**Affiliations:** ^1^Shandong Provincial Center for Disease Control and Prevention, Jinan, China; ^2^Department of Veterinary Medicine, College of Veterinary Medicine, Zhejiang University, Hangzhou, China; ^3^Shandong Medical College, Jinan, China; ^4^Linyi Center for Disease Control and Prevention, Linyi, China; ^5^Institute for Veterinary Food Science, Justus-Liebig-University Giessen, Giessen, Germany; ^6^State Key Laboratory for Diagnosis and Treatment of Infectious Diseases, National Clinical Research Center for Infectious Diseases, National Medical Center for Infectious Diseases, The First Affiliated Hospital, College of Medicine, Zhejiang University, Hangzhou, China; ^7^The Hainan Institute of Zhejiang University, Sanya, China

**Keywords:** *Salmonella* Typhi, outbreak, typhoid fever, drinking water, genomics

An author name was incorrectly spelled as “Bing Hu”. The correct spelling is “Bin Hu”.

The authors apologize for this error and state that this does not change the scientific conclusions of the article in any way. The original article has been updated.

## Publisher's Note

All claims expressed in this article are solely those of the authors and do not necessarily represent those of their affiliated organizations, or those of the publisher, the editors and the reviewers. Any product that may be evaluated in this article, or claim that may be made by its manufacturer, is not guaranteed or endorsed by the publisher.

