# Supplementary figures and images for: Genomic Investigation Reveals a Community Typhoid Outbreak Caused by Contaminated Drinking Water in China, 2016
Source: Front Med (Lausanne). 2022 Mar 1;9:753085. doi: 10.3389/fmed.2022.753085 (PMC8925297; doi:10.3389/fmed.2022.753085)

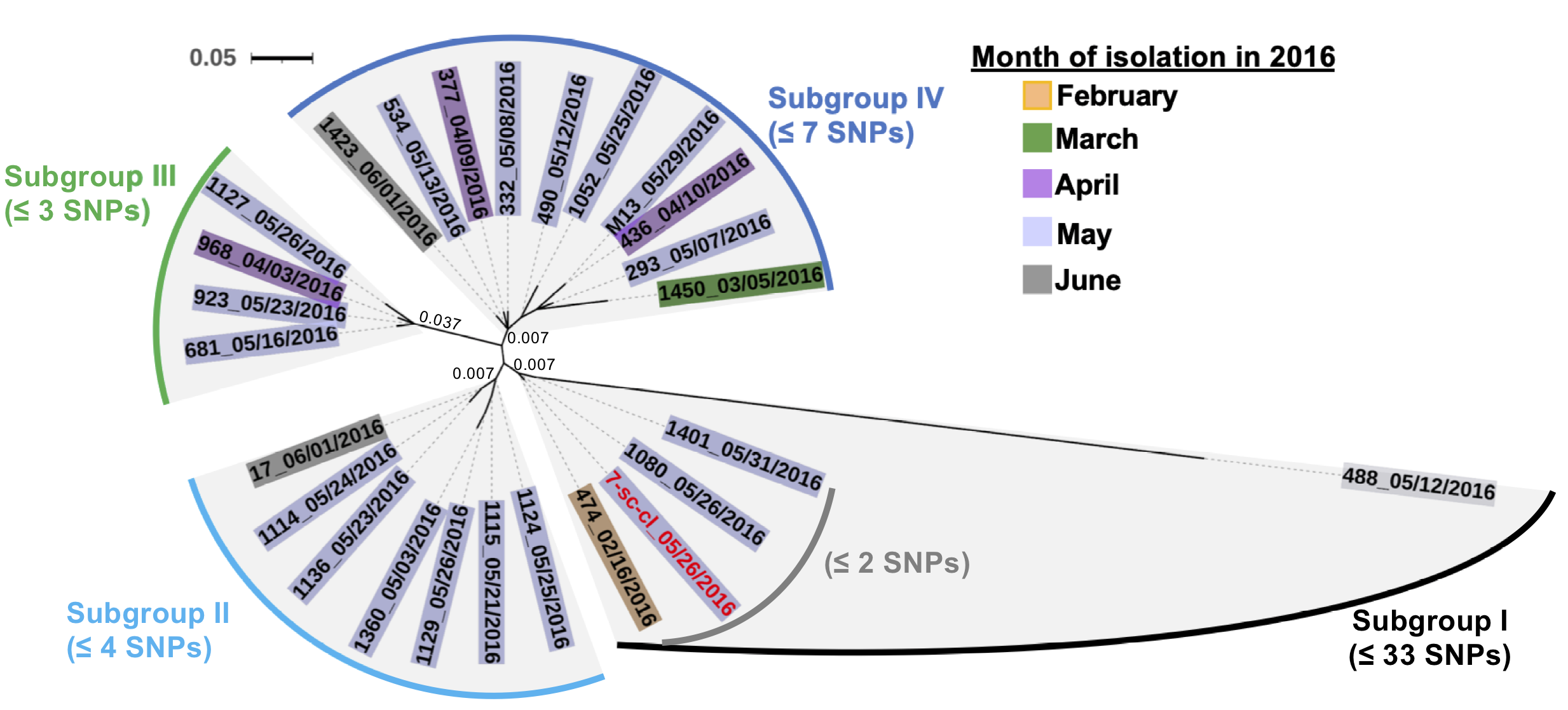

Supplement: Supplementary Figure S1 — Unrooted phylogenetic tree of the examined isolates from this study. The maximum-likelihood phylogenetic tree was generated using Parsnp and visualized using iTOL. [file Image_1.TIFF]

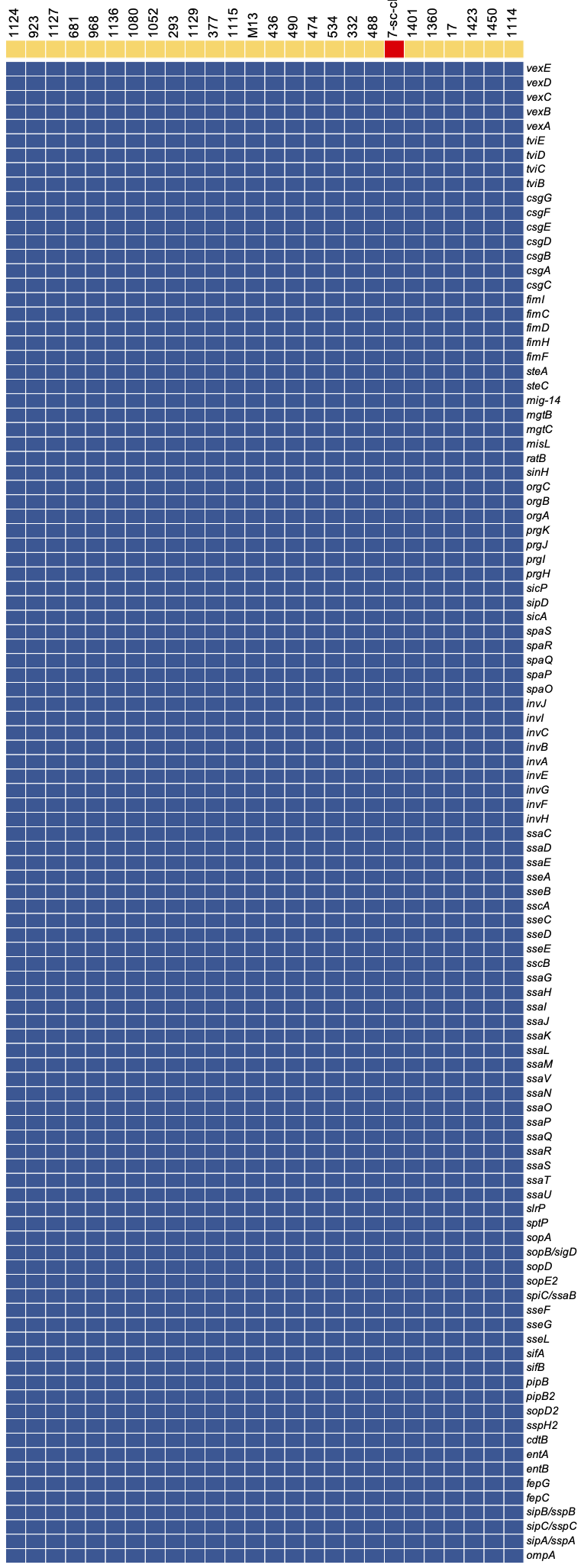

Supplement: Supplementary Figure S2 — Virulence factor gene profiles of 26 S. Typhi isolates. The orange and red blocks indicate the isolates are from patients' stools and water samples, respectively. The blue blocks indicate the presence of the virulence genes. [file Image_2.TIFF]
